# Effects of *Helicobacter pylori* eradication on serum lipid levels

**DOI:** 10.3164/jcbn.17-88

**Published:** 2018-01-27

**Authors:** Kyoichi Adachi, Tomoko Mishiro, Takashi Toda, Naomi Kano, Harumi Fujihara, Yuko Mishima, Atsuko Konishi, Mariko Mochida, Kazuko Takahashi, Yoshikazu Kinoshita

**Affiliations:** 1 Health Center, Shimane Environment and Health Public Corporation, Koshibara 1-4-6, Matsue, Shimane 690-0012, Japan; 2 Clinical Laboratory, Shimane Environment and Health Public Corporation, Matsue, Shimane 690-0012, Japan; 3 Second Department of Internal Medicine, Shimane University Faculty of Medicine, Izumo, Shimane 693-8501, Japan

**Keywords:** *Helicobacter pylori*, eradication, serum lipids, HDL-cholesterol, LDL/HDL-cholesterol ratio

## Abstract

The purpose was to clarify the effects of *Helicobacter pylori* (*H. pylori*) eradication on the changes in serum lipid levels by comparing subjects with and without continuous *H. pylori* infection. The study subjects were 774 individuals (males 536, females 238, mean age 52.6 years) who visited between April 2013 and March 2016 for annual medical checkups. Serum total cholesterol, high-density lipoprotein cholesterol (HDLC), low-density lipoprotein cholesterol (LDLC), and triglyceride levels, and LDLC/HDLC ratio were compared between the subjects with and without *H. pylori* infection, as well as those with *H. pylori* eradication subjects. The HDLC level in the *H. pylori*-positive group was significantly lower as compared to the *H. pylori*-negative group. The serum level of HDLC in subjects with successful eradication of *H. pylori* tended to be higher, while the serum levels of total cholesterol, LDLC, and triglycerides tended to be lower in comparison to subjects with continuous *H. pylori* infection. In addition, the LDLC/HDLC ratio in the *H. pylori*-positive group was significantly higher than that in the *H. pylori*-negative group, and successful *H. pylori* eradication tended to reduce that ratio. In conclusion, successful eradication of *H. pylori* may have favorable effects on lipid metabolism.

## Introduction

Cardiovascular disease is one of the most important life-threating diseases in developed countries. Several bacterial and viral infections, such as Chlamydia and cytomegalovirus, have been repeatedly demonstrated to have effects on lipid metabolism and arteriosclerosis progression, well-known risk factors for the onset of cardiovascular disease.^(1–4)^ Infection with *Helicobacter pylori* (*H. pylori*), an important pathogen involved in several gastroduodenal diseases, has also been suggested to correlate with the onset of cardiovascular disease due to its effects on changes in lipid metabolism.^(1,5–8)^ Previous reports in Western countries have shown that *H. pylori* infection leads to increased total cholesterol and triglyceride levels, while low high-density lipoprotein cholesterol (HDLC) and elevated low-density lipoprotein cholesterol (LDLC) have been reported in *H. pylori*-positive subjects in Japan and other Asian countries.^(7–13)^ Therefore, the influence of *H. pylori* infection on lipid metabolism may differ among different ethnic groups.

Eradication of *H. pylori* was reported to increase body weight and alter serum lipids.^(14–16)^ On the other hand, the effects of *H. pylori* eradication therapy on the changes in serum level of lipids has not been fully determined in subjects with and without continuous *H. pylori* infection. In February 2013, the national health insurance system of Japan began coverage for *H. pylori* eradication therapy to treat *H. pylori*-associated chronic gastritis, which is closely associated with development of atrophic gastritis and gastric cancer.^(17)^ Since that time, there has been a rapid increase of patients in Japan undergoing eradication therapy for *H. pylori* infection. Thus, the influence of that therapy on lipid metabolism is considered to be an important issue to predict the risk of arteriosclerosis progression and cardiovascular disease onset. We investigated the influence of *H. pylori* eradication on lipid metabolism in healthy subjects who came to our medical center for annual medical check-ups.

## Subjects and Methods

The study subjects were individuals who visited the Health Center of Shimane Environment and Health Public Corporation for annual detailed medical checkup examinations between April 2013 and March 2016. The majority were socially active and productive, and considered to be socioeconomically middle class. Those taking medication for hyperlipidemia were excluded to eliminate the influence of medication on serum lipid parameters. A total of 774 subjects (males 536, females 238, mean age 52.6 years) underwent an examination to determine the presence of *H. pylori* infection between April 2013 and March 2014, and then visited our medical center again for annual medical checkups twice between April 2014 and March 2016. A precise medical history was noted and a physical examination was carried out in each visits, and hematological and biochemical blood tests of samples obtained after overnight fasting were performed at the laboratory of Shimane Environment and Health Public Corporation. Thus, all 774 study subjects underwent biochemical blood tests for serum lipids a total of 3 times between April 2013 and March 2016. The status of *H. pylori* infection was determined by detection of the antibody for *H. pylori *in urine or serum using the Rapirun immunochromatography method (Otsuka Pharmaceutical, Tokushima, Japan) and MinuteRead anti-PYLORI (Institute of Immunology Co., Ltd., Tokyo), respectively. All subjects also underwent upper GI endoscopic examinations by licensed three experienced endoscopists at the time of each medical checkup. Presence or absence of *H. pylori* infection was also evaluated by several endoscopic findings, such as presence or absence of gastric mucosal atrophy, diffuse redness, mucosal swelling, sticky mucous and enlarged folds and regular arrangement of collecting venules at angular portion according to the previous reports.^(18–21)^ When the inconsistency was observed between the result of *H. pylori* antibody test and the endoscopic findings, we recommended that the patient undergo *H. pylori *stool antigen test or urea breath test.

We advised all subjects with *H. pylori *infection to receive eradication therapy for *H. pylori* in other medical centers, since our institute is a medical center only for annual detailed medical checkup examinations and we do not perform eradication therapy at our institute.

Performance of eradication therapy for *H. pylori* and successful eradication between the annual medical check-ups were determined based on the precise medical history of each subject obtained by a public health nurse. Almost all *H. pylori*-positive subjects in second and third visit periods did not undergo eradication therapy for *H. pylori*, although we advised to perform eradication therapy. Successful eradication therapy for *H. pylori* was determined by the result of urea breath test or stool antigen test. Those without successful eradication were included in the group with *H. pylori* infection, even when they had received eradication therapy. When eradication therapy was not confirmed as successful, we recommended that the patient undergo an *H. pylori* stool antigen test at our institution. Successful eradication was also confirmed by changes in gastric mucosal findings obtained in an upper GI endoscopic examination, which was performed for all subjects. Gastric mucosal findings for assessment of successful eradication were the presence of map-like redness or depressed patchy redness, as well as absence of diffuse redness, mucosal swelling, sticky mucous and enlarged folds.^(18–22)^ When the gastric mucosal findings were not changed in comparison with those prior to eradication therapy, we diagnosed the subject as positive for *H. pylori* infection regardless of eradication therapy. All of the endoscopic images from each subject were simultaneously reviewed by three licensed endoscopists, and the diagnosis of each endoscopic finding was made. If there were any inconsistencies in the reading of the endoscopic images between the three endoscopists, the final diagnosis was decided by one endoscopist (K.A.).

The factors investigated in this study were gender, age, body mass index (BMI), and smoking and drinking (≥50 ml alcohol/day) habits, as well as serum levels of total cholesterol, HDLC, LDLC and triglycerides. Serum total cholesterol, HDLC, LDLC, triglycerides and LDLC/HDLC ratio were compared between the *H. pylori*-positive and -negative groups before and after adjusting for covariant factors (gender, age, BMI, smoking, drinking) using analysis of covariance. These parameters were compared between those groups using findings obtained at the first examination (April 2013 to March 2014), and then among *H. pylori*-positive, -negative, and post-eradication subjects using findings obtained at the second (April 2014 to March 2015) and third (April 2015 to March 2016,) examinations. In addition, the numbers of cases, who showed total cholesterol ≥220 mg/dl, HDLC ≤39 mg/dl, LDLC ≥140 mg/dl, and triglycerides ≥150 mg/dl, were analyzed.

Statistical analyses were performed using chi-squared teat and Mann-Whitney *U* test to compare among the groups, and an analysis of covariance was used to compare between the data of the groups for the adjustment for covariant factors. Wilcoxon signed-rank test was used for analysis of time-course changes in each group. Stat View 5.0 (Abacus Concepts Inc., Berkeley, CA) for Macintosh and the SPSS statistical package (ver. 19J for Windows; SPSS, Chicago, IL) were used to perform statistical analyses. Differences at *p*<0.05 were considered to be statistically significant.

This study was performed in accordance with the Declaration of Helsinki and approved by the Ethics Committee of Shimane Institute of Health Science.

## Results

**Fig. 1:**
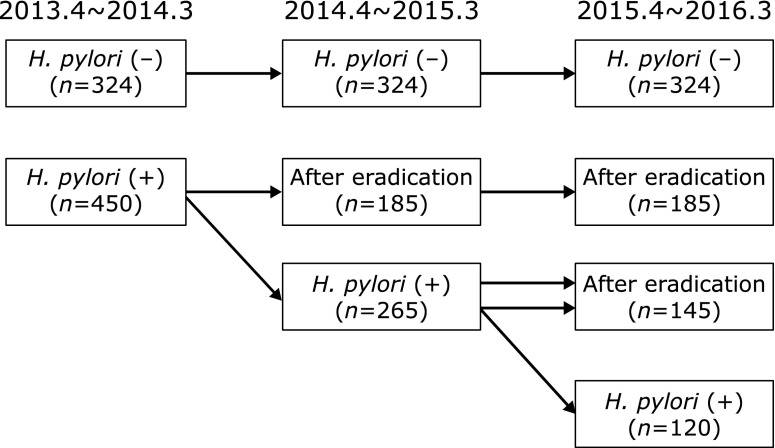
Time course changes in study subjects with and without *H. pylori* infection.

The 774 subjects who participated in the first visit between April 2013 and March 2014 were divided into *H. pylori*-negative (*n* = 324, 41.9%) and -positive (*n* = 450, 58.1%) groups. Subjects with *H. pylori* infection were older than those without *H. pylori* infection. During the second visit period (April 2014 to March 2015), 185 of the *H. pylori*-positive subjects visited for annual detailed medical checkup after successful eradication of *H. pylori*, while the remaining 265 showed continuing infection due to no performance of eradication therapy or unsuccessful eradication. At the third visit (April 2015 to March 2016), 145 of those 265 subjects positive for infection at the second visit showed successful eradication of *H. pylori*, while the remaining 120 remained infected. Subjects without *H. pylori* eradication tended to be male and younger than those who showed eradication (Table 1, 2 and 3 and Fig. 1). BMI in cases with successful eradication of *H. pylori* tended to be lower than that in cases with continuous *H. pylori* infection.

Serum levels of total cholesterol, HDLC, LDLC and triglycerides before and after adjusting for confounding factors (age, sex, BMI, smoking, drinking) in all subjects are shown in Table 1, 2 and 3. HDLC in the *H. pylori*-positive group was significantly lower as compared to the *H. pylori*-negative group at the first and second visits, even after adjusting for confounding factors, and the same tendency was observed at the third visit. Furthermore, the serum level of HDLC in subjects with successful eradication of *H. pylori* tended to be higher and the serum levels of total cholesterol, LDLC, and triglyceride tended to be lower in comparison to subjects with continuous *H. pylori* infection. The numbers of cases, who showed total cholesterol ≥220 mg/dl, LDLC ≥140 mg/dl, and triglycerides ≥150 mg/dl, tended to be higher in the subjects without *H. pylori* eradication in comparison with those with successful eradication and without *H. pylori* infection (Table 2 and 3).

**Fig. 2:**
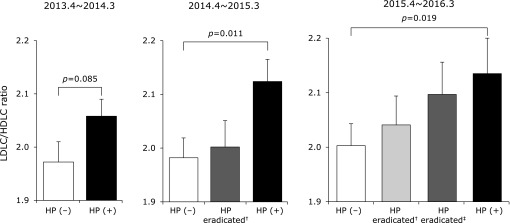
LDL-cholesterol (LDLC)/HDL-cholesterol (HDLC) ratio in subjects with and without *H. pylori* (HP) infection. Data are expressed as the mean ± SE after adjustments for covariant factors (gender, age, BMI, smoking, drinking). ^†^Eradication shown before second visit (April 2014 to March 2015). ^‡^Eradication shown before third visit (April 2015 to March 2016).

**Fig. 3:**
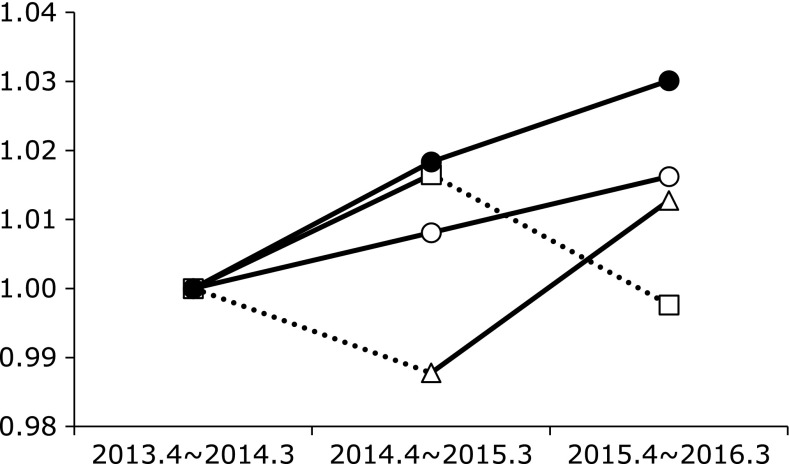
Time-course changes of LDL-cholesterol (LDLC)/HDL-cholesterol (HDLC) ratios of 4 groups. Data was express as the ratio to the data of first visit (April 2013 to March 2014). ◯: *H. pylori*-negative subjects, ●: *H. pylori*-positive subjects, △: *H. pylori*-eradicated subjects (successful eradication before 2nd visit), □: *H. pylori*-eradicated subjects (successful eradication before 3rd visit). There was no significant difference in time-course changes of all groups.

The LDLC/HDLC ratio was significantly greater in the *H. pylori*-positive as compared to the *H. pylori*-negative subjects at the second and third visits. In addition, successful eradication of *H. pylori* tended to reduce the LDLC/HDLC ratio in comparison to subjects with continuous *H. pylori* infection, though that ratio in subjects with successful eradication tended to be higher than in those negative for *H. pylori* (Fig. 2). The time-course changes in *H. pylori*-negative, positive and eradicated groups showed that LDLC/HDLC ratio tended to decrease after successful eradication for *H. pylori*, although the LDLC/HDLC ratio increased with aging in all groups (Fig. 3).

## Discussion

*H. pylori* infection is known to be related to the onset of several gastroduodenal diseases, including gastritis, peptic ulcer disease, gastric cancer and low-grade B cell lymphoma in gastric mucosa-associated-lymphoid tissue.^(23–26)^ Bacterial and viral infections were shown to be associated with increased serum levels of fibrinogen and lipids, well-known risk factors for cardiovascular disease.^(1–4)^ Also, persistent low-grade inflammation induced by *H. pylori* infection was reported to produce pro-inflammatory cytokines, such as C reactive protein, and interleukin (IL)-6 and IL-8, which are considered to have important effects on lipid metabolism and arteriosclerosis progression.^(27)^ It has been demonstrated that serum cholesterol and triglyceride levels are significantly higher in Western subjects infected with *H. pylori*.^(7–8)^ On the other hand, in Japanese, HDLC level has been reported to be significantly different between *H. pylori*-positive and -negative individuals after adjustments for gender, age, BMI, and drinking and smoking status, which was confirmed by the present results.^(9–11)^

Successful eradication of *H. pylori* infection has been demonstrated to not only prevent several gastro-duodenal diseases, but also improve extra-digestive diseases, such as idiopathic thrombocytopenia and iron deficiency anemia.^(28,29)^ Therefore, *H. pylori* eradication has been repeatedly recommended in reports published throughout the world. In Japan, national health insurance coverage began in February 2013 for all patients with *H. pylori*-associated chronic gastritis and eradication therapy has quickly become widespread.^(17)^ Successful *H. pylori* eradication may also improve persistent low-grade gastric inflammation, as well as have a favorable effect on lipid metabolism by inhibiting the production of pro-inflammatory cytokines and their release from gastric mucosa. However, the influence of *H. pylori* eradication therapy on the changes of lipid metabolism in subjects with and without continuous *H. pylori* infection has not been fully investigated. In the present study, we analyzed the effects of therapy for eradication of *H. pylori* infection in regard to the changes in serum levels of total cholesterol, HDLC, LDLC and triglycerides, as well as LDLC/HDLC ratio in healthy Japanese subjects who attended our medical center for annual medical check-ups.

Increased body weight has been repeatedly observed following successful eradication of *H. pylori*.^(14–16)^ BMI in cases with successful eradication was not higher than that with continuously positive of *H. pylori* infection in this study, which was performed in mainly healthy asymptomatic subjects visited for annual medical checkups. Eradication therapy in previous studies was mainly performed in cases with peptic ulcer diseases.^(14,15)^ Thus, alterations in body weight after successful eradication might be different among the cases with different upper gastro-intestinal disease conditions. Alterations in body weight should be considered when analyzing the influence of *H. pylori* eradication on changes in serum lipid levels, since body weight increase is a well-known risk factor related to a worsened serum lipid level. However, the change of body weight after eradication for *H. pylori* was not adjusted in previous studies which demonstrated the increment of serum levels of total cholesterol and triglyceride after eradication.^(12,13,15,30,31)^ The present results demonstrated that eradication of *H. pylori* has effects on the changes of serum lipids even after adjusting for covariant factors, including BMI, and smoking and drinking habits. The serum level of HDLC in our subjects with successful eradication tended to be higher, while the serum levels of total cholesterol, LDLC and triglycerides tended to be lower in comparison to subjects with continuous *H. pylori* infection. The differences in serum lipid levels between the cases with and without successful eradication might be small. However, the cases with high levels of total cholesterol, LDLC and triglycerides were frequently observed in cases with continuous *H. pylori* infection. In addition, eradication of *H. pylori* tended to reduce the LDLC/HDLC ratio as compared to subjects with continuous infection, and LDLC/HDLC ratio tended to decrease after successful eradication for *H. pylori*. It has been demonstrated that the increment of serum HDLC level after successful eradication for *H. pylori,* although the changes of LDLC were not significantly decreased.^(12,30,31)^ LDLC/HDLC ratio is reported to be a good parameter for estimating the risk of future cardiovascular events.^(32–37)^ Therefore, the change in serum lipid levels after successful eradication of *H. pylori* infection is considered to have an inhibitory effect on arteriosclerosis progression and onset of cardiovascular disease.

This study has several limitations. The subjects are not representative of the general population, as they were individuals who voluntarily visited our medical center for annual check-ups, with the numbers of young and elderly subjects relatively small. In addition, we did not investigate the degree of gastritis and the severity of gastric mucosal atrophy in this study. The degree of gastritis and the severity of gastric mucosal atrophy were considered to influence the lipid metabolisms, since those are related to the production of pro-inflammatory cytokines and ghrelin from the gastric mucosa.^(27,38,39)^ Therefore, the influence of degree of gastritis and severity of gastric mucosal atrophy on the lipid metabolism should be investigated before and after eradication for *H. pylori* in future studies. The follow-up period after eradication of *H. pylori* was 2 years, too short to investigate the progression of arteriosclerosis or occurrence of cardiovascular events. To our knowledge, long-term effect of eradication for *H. pylori* on the lipids metabolism and the occurrence of cardiovascular disease has not been previously demonstrated.^(12–15,30,31)^ The effects of *H. pylori* on lipid metabolism may be induced over a long period, since that infection mainly occurs during childhood.^(17,40)^ Therefore, further large-scale long-term prospective studies are recommended to confirm the influence of eradication of *H. pylori* infection on lipid metabolism, as well as progression of arteriosclerosis and occurrence of cardiovascular disease.

In conclusion, successful eradication of *H. pylori* may have favorable effects on time-course changes in serum lipid levels, such as total cholesterol, HDLC, LDLC and triglycerides, as well as LDLC/HDLC ratio.

## Figures and Tables

**Table 1:** *H. pylori* infection status and serum lipid parameters at first visit (April 2013 to March 2014)

*H. pylori* status	Negative	Positive
Gender (male/female)	218/106	318/132
Age in years	51.1 ± 0.50	53.7 ± 0.4**
BMI	22.8 ± 0.2	22.6 ± 0.1
T cho. (mg/dl)	205.8 ± 1.7	203.0 ± 1.5
Adjusted value (mg/dl)	206.2 ± 1.7	202.7 ± 1.5
High T cho.	106 (32.7)	141 (31.3)
HDLC (mg/dl)	70.0 ± 1.0	67.2 ± 0.8*
Adjusted value (mg/dl)	70.2 ± 0.9	67.0 ± 0.7**
Low HDLC	8 (2.5)	11 (2.4)
LDLC (mg/dl)	127.8 ± 1.6	128.3 ± 1.4
Adjusted value (mg/dl)	128.1 ± 1.6	128.1 ± 1.4
High LDLC	108 (33.3)	164 (36.4)
TG (mg/dl)	109.2 ± 4.0	104.8 ± 3.2
Adjusted value (mg/dl)	109.5 ± 3.6	104.7 ± 3.1
High TG	57 (17.6)	65 (14.4)

Data was expressed by mean ± SE. BMI: body mass index, T cho.: total cholesterol, Adjusted value: Value adjusted for covariant factors (gender, age, BMI, smoking, drinking). High T cho.: Total cholesterol ≥220 mg/dl. HDLC: HDL-cholesterol. Low HDLC: HDL-cholesterol ≤39 mg/dl. LDLC: LDL-cholesterol. High LDLC: LDL-cholesterol ≥140 mg/dl. TG: triglycerides, High TG: triglycerides ≥150 mg/dl. Numbers in Parenthesis indicate percentage. *p<0.05 and **p<0.01 in comparison with *H. pylori-*negative subjects.

**Table 2:** *H. pylori* infection status and serum lipid parameters at second visit (April 2014 to March 2015)

*H. pylori* status	Negative	Eradication before 2nd visit	Positive
Gender (male/female)	218/106	117/68	201/64*
Age in years	52.1 ± 0.5	55.6 ± 0.6*	54.1 ± 0.5*
BMI	22.8 ± 0.2	22.1 ± 0.2	22.9 ± 0.2^#^
T cho. (mg/dl)	206.2 ± 1.7	204.8 ± 2.5	207.1 ± 2.0
Adjusted value (mg/dl)	206.5 ± 1.7	203.7 ± 2.3	207.5 ± 1.9
High T cho.	97 (29.9)	61 (33.0)	93 (35.1)
HDLC (mg/dl)	68.2 ± 1.0	68.5 ± 1.3	64.3 ± 1.0*^,#^
Adjusted value (mg/dl)	68.4 ± 0.8	66.6 ± 1.1	65.3 ± 0.9*
Low HDLC	9 (2.8)	2 (1.1)	11 (4.2)
LDLC (mg/dl)	125.1 ± 1.6	124.4 ± 2.1	128.8 ± 1.8
Adjusted value (mg/dl)	125.1 ± 1.5	124.4 ± 2.1	128.8 ± 1.7
High LDLC	94 (29.0)	59 (31.9)	88 (33.2)
TG (mg/dl)	106.6 ± 4.2	97.0 ± 4.6	113.4 ± 4.3*^,#^
Adjusted value (mg/dl)	106.5 ± 3.7	103.0 ± 4.9	109.3 ± 4.1
High TG	53 (16.4)	19 (10.2)	53 (20.0)^#^

Data was expressed by mean ± SE. BMI: body mass index, T cho.: total cholesterol, Adjusted value: Value adjusted for covariant factors (gender, age, BMI, smoking, drinking). High T cho.: Total cholesterol ≥220 mg/dl. HDLC: HDL-cholesterol. Low HDLC: HDL-cholesterol ≤39 mg/dl. LDLC: LDL-cholesterol. High LDLC: LDL-cholesterol ≥140 mg/dl. TG: triglycerides, High TG: triglycerides ≥150 mg/dl. Numbers in Parenthesis indicate percentage. *p<0.05 in comparison with *H. pylori*-negative subjects. ^#^*p*<0.05 in comparison with subjects showing eradication before second visit.

**Table 3:** *H. pylori* infection status and serum lipid parameters at third visit (April 2015 to March 2016)

*H. pylori* status	Negative	Eradication before 2nd visit	Eradication before 3rd visit	Positive
Gender (male/female)	218/106	117/68	107/38	94/26*^,#^
Age in years	53.1 ± 0.5	56.6 ± 0.6*	55.3 ± 0.7*	54.8 ± 0.8^#^
BMI	22.7 ± 0.2	22.1 ± 0.2	22.8 ± 0.3	22.9 ± 0.3^#^
T cho. (mg/dl)	206.9 ± 1.7	208.2 ± 2.5	207.8 ± 2.8	209.8 ± 3.1
Adjusted value (mg/dl)	207.3 ± 1.8	206.9 ± 2.4	207.8 ± 2.7	210.7 ± 3.0
High T cho.	104 (32.1)	64 (34.6)	53 (36.6)	44 (36.7)
HDLC (mg/dl)	68.3 ± 1.0	69.0 ± 1.3	65.9 ± 1.5	65.1 ± 1.6^#^
Adjusted value (mg/dl)	68.4 ± 0.9	67.3 ± 1.2	66.4 ± 1.3	66.7 ± 1.5
Low HDLC	8 (2.5)	4 (2.2)	4 (2.8)	3 (2.5)
LDLC (mg/dl)	125.7 ± 1.6	127.5 ± 2.2	129.2 ± 2.5	130.8 ± 2.9
Adjusted value (mg/dl)	125.8 ± 1.6	127.3 ± 2.1	129.1 ± 2.4	131.0 ± 2.7
High LDLC	90 (27.8)	56 (30.3)	49 (33.8)	44 (36.7)
TG (mg/dl)	100.6 ± 3.3	94.6 ± 4.2	104.6 ± 5.3	111.9 ± 5.6*^,#^
Adjusted value (mg/dl)	101.3 ± 3.1	99.3 ± 4.1	102.3 ± 4.6	105.6 ± 5.0
High TG	46 (14.2)	22 (11.9)	25 (17.2)	24 (20.0)

Data was expressed by mean ± SE. BMI: body mass index, T cho.: total cholesterol, Adjusted value: Value adjusted for covariant factors (gender, age, BMI, smoking, drinking). High T cho.: Total cholesterol ≥220 mg/dl. HDLC: HDL-cholesterol. Low HDLC: HDL-cholesterol ≤39 mg/dl. LDLC: LDL-cholesterol. High LDLC: LDL-cholesterol ≥140 mg/dl. TG: triglycerides, High TG: triglycerides ≥150 mg/dl. Numbers in parenthesis indicate percentage. *p<0.05 in comparison with *H. pylori*-negative subjects. ^#^*p*<0.05 in comparison with the subjects showing eradication before second visit.
